# Characterization of an Arginine Decarboxylase from *Streptococcus pneumoniae* by Ultrahigh-Performance Liquid Chromatography–Tandem Mass Spectrometry

**DOI:** 10.3390/biom14040463

**Published:** 2024-04-10

**Authors:** Jung Hwa Lee, Moses B. Ayoola, Leslie A. Shack, Edwin Swiatlo, Bindu Nanduri

**Affiliations:** 1 Department of Comparative Biomedical Sciences, College of Veterinary Medicine, Mississippi State University, Starkville, MS 39762, USA; 2 Section of Infectious Diseases, Southeast Louisiana Veterans Health Care System, New Orleans, LA 70112, USA

**Keywords:** *Streptococcus pneumoniae*, polyamine, SP_0166, arginine decarboxylase, DFMA, DFMO

## Abstract

Polyamines are polycations derived from amino acids that play an important role in proliferation and growth in almost all living cells. In *Streptococcus pneumoniae* (the pneumococcus), modulation of polyamine metabolism not only plays an important regulatory role in central metabolism, but also impacts virulence factors such as the capsule and stress responses that affect survival in the host. However, functional annotation of enzymes from the polyamine biosynthesis pathways in the pneumococcus is based predominantly on computational prediction. In this study, we cloned SP_0166, predicted to be a pyridoxal-dependent decarboxylase, from the Orn/Lys/Arg family pathway in *S. pneumoniae* TIGR4 and expressed and purified the recombinant protein. We performed biochemical characterization of the recombinant SP_0166 and confirmed the substrate specificity. For polyamine analysis, we developed a simultaneous quantitative method using hydrophilic interaction liquid chromatography (HILIC)-based liquid chromatography–tandem mass spectrometry (LC–MS/MS) without derivatization. SP_0166 has apparent *Km*, *kcat*, and *kcat*/*Km* values of 11.3 mM, 715,053 min^−1^, and 63,218 min^−1^ mM^−1^, respectively, with arginine as a substrate at pH 7.5. We carried out inhibition studies of SP_0166 enzymatic activity with arginine as a substrate using chemical inhibitors DFMO and DFMA. DFMO is an irreversible inhibitor of ornithine decarboxylase activity, while DFMA inhibits arginine decarboxylase activity. Our findings confirm that SP_0166 is inhibited by DFMA and DFMO, impacting agmatine production. The use of arginine as a substrate revealed that the synthesis of putrescine by agmatinase and *N*-carbamoylputrescine by agmatine deiminase were both affected and inhibited by DFMA. This study provides experimental validation that SP_0166 is an arginine decarboxylase in pneumococci.

## 1. Introduction

*Streptococcus pneumoniae*, commonly known as the pneumococcus, is a Gram-positive facultative anaerobe [1]; it is the primary cause of mortality in children under 5 years of age globally and contributes significantly to fatalities across all age groups [2,3]. Based on the unique capsular polysaccharide (CPS) structure, about 100 pneumococcal serotypes are known. Current vaccines target CPS from serotypes most frequently isolated from human infections [4]. Among its arsenal of virulence factors, the capsule stands out as a principal determinant, contributing to immune evasion and survival in the host. In addition to the capsule, *S. pneumoniae* employs various virulence factors, including adhesins (such as choline-binding protein A, neuraminidase A, and LPXTG proteins), cytotoxins (pneumolysin), and immune-evasive proteins (pneumococcal surface protein A, plasmin-binding, and fibronectin-binding proteins), all of which play crucial roles in adhesion, colonization, cytotoxicity, and evasion of the host immune system [5]. Recently, polyamines have been associated with pneumococcal virulence, and polyamine metabolism in pneumococci serves as an important regulator of central metabolism [6]. Polyamines represent a group of aliphatic polycationic hydrocarbons mostly associated with RNA that play critical roles in diverse biological processes, including cell growth, transcription, and translation [7,8,9]. The primary polyamines encompass putrescine, spermidine, spermine, and cadaverine.

Within the capsular serotypes of *S. pneumoniae* linked to invasive diseases, the conservation of putative lysine decarboxylase (CadA), spermidine synthase (SpeE), and substrate binding protein of the polyamine transporter protein (PotD) has been confirmed through analysis, exhibiting more than 99% identity across sequenced pneumococcal genomes, and the presence of these genes is shown to be important for in vivo fitness [10]. Initially, our research described the importance of CadA in virulent pneumococcal serotype 4 capsule biosynthesis and its regulatory role in the interplay between stress response and capsule synthesis [11,12]. However, our subsequent studies established that CadA is actually an arginine decarboxylase rather than a lysine decarboxylase [13]. This discrepancy highlights the issues with the current annotation of polyamine metabolism genes in public databases which are primarily based on computational prediction without experimental validation. Current knowledge of polyamine synthesis derives from inferences from *E. coli*, emphasizing the need for alternative annotations specific to genetically divergent organisms. The primary route for bacterial polyamine synthesis involves the decarboxylation of ornithine to putrescine, a reaction catalyzed by ornithine decarboxylase (ODC), with pyridoxal 5′-phosphate (PLP) serving as a cofactor [14]. Additional routes of putrescine synthesis include a two-step process from arginine, utilizing arginine decarboxylase (ADC) and agmatinase with agmatine as an intermediate, as well as a three-step process involving ADC, agmatine deiminase, and *N*-carbamoylputrescine amidohydrolase, with agmatine and *N*-carbamoylputrescine as intermediates [7,14]. Subsequently, spermidine and spermine are produced from putrescine through the sequential actions of spermidine synthase and spermine synthase, respectively. In reversible reactions, spermidine/spermine acetyltransferase can acetylate both spermine and spermidine, while polyamine oxidases convert them back to spermidine and putrescine, respectively. Additionally, a distinct pathway involves the decarboxylation of lysine to cadaverine, catalyzed by lysine decarboxylase.

Ornithine decarboxylation is the rate-limiting step in polyamine biosynthesis [15]. Treatment with the irreversible ODC inhibitor difluoromethylornithine (DFMO) is known to deplete cellular levels of putrescine, spermidine, and agmatine [16,17]. DFMO, acting as a suicide inhibitor, undergoes decarboxylation by ODC akin to ornithine. This process generates a reactive intermediate that forms a stable covalent bond with surrounding groups, resulting in ODC inactivation [18]. DFMO is shown to be an effective chemotherapeutic agent, particularly in hyperproliferative diseases such as cancer [19,20,21]. It is noteworthy that DFMO not only targets ODC but also inhibits ADC activity [16,22]. Difluoromethyl arginine (DFMA) [23], employed as an ADC inhibitor in various microorganisms and plants, leads to reduced agmatine and putrescine levels in diverse organisms [24,25,26]. Genes encoding enzymes for the conversion of ornithine to putrescine and lysine to cadaverine in TIGR4 have not been identified. Current genome annotation of SP_0166 is stated as “pyridoxal-dependent decarboxylase, Orn/Lys/Arg family” with only basic annotation score of 1 out of 5 (https://www.uniprot.org/uniprotkb/A0A0H2UNA7/entry, accessed on 5 March 2024) [27]. Based on this description, SP_0166 has the potential to utilize ornithine, lysine, and arginine. This study is designed to determine the precise catalytic role of SP_0166 in pneumococcal polyamine biosynthesis. Here, we utilized liquid chromatography–tandem mass spectrometry (LC–MS/MS) employing a hydrophilic interaction liquid chromatography (HILIC) to characterize the enzymatic activity of SP_0166. We also measured the kinetic parameters with arginine, lysine, and ornithine substrates and the effect of chemical inhibitors.

## 2. Materials and Methods

### 2.1. Cloning, Expression, and Purification of SP_0166

The *SP_0166* gene locus in TIGR4 encodes a protein of 368 amino acids with a predicted molecular weight of ~43 kDa. *SP_0166* was amplified from chromosomal DNA of TIGR4 using primers with BamHI and XhoI restriction sites (Table 1). The PCR product was cloned into the pET-28a (+) vector (MilliporeSigma, Burlington, MA, USA) with a 6x -His tag at the C-terminus. The resulting recombinant expression vector was transformed into the *E. coli* strain BL21 (DE3), which was grown in Terrific broth (Thermo Fisher Scientific, Waltham, MA, USA) containing 30 µg/mL kanamycin and 3% ethanol at 37 °C to an optical density of 1.1 at 600 nm and induced with 1.0 mM 1-thio-β-D-galactopyranoside (IPTG). Five hours post-induction, cells were harvested by centrifugation at 5000× *g* for 10 min at 4 °C and the cell pellet was stored at −20 °C until further use. The frozen pellet was thawed on ice and resuspended in B-PER Reagent buffer (Thermo Fisher Scientific, Waltham, MA, USA) at 4 mL/g of pellet, with 2 µL of benzonase (Sigma-Aldrich, St. Louis, MO, USA) and 10 µL/mL protease inhibitor (Thermo Fisher Scientific, Waltham, MA, USA), and incubated for 15 min at room temperature.

The cell debris was removed by centrifugation at 15,000× *g* for 5 min and the lysate was loaded onto a HisPur Cobalt Spin Column (Thermo Fisher Scientific, Waltham, MA, USA). After washing with equilibration/wash buffer containing 100 mM imidazole, bound proteins were eluted with 500 mM imidazole in elution buffer. The purified protein was desalted using a Sephadex G-25 PD-10 column (GE Healthcare, Chicago, IL, USA) equilibrated with PBS. Purified protein was evaluated by sodium dodecyl sulfate polyacrylamide gel electrophoresis (SDS-PAGE) and staining with Coomassie Brilliant Blue R-250 (Bio-Rad, Hercules, CA, USA). Protein estimation was performed using the BCA method with Pierce BCA Protein Assay kit (Thermo Fisher Scientific, Waltham, MA, USA).

### 2.2. Sample Preparation to Determine Substrate Specificity

**Figure 1:**
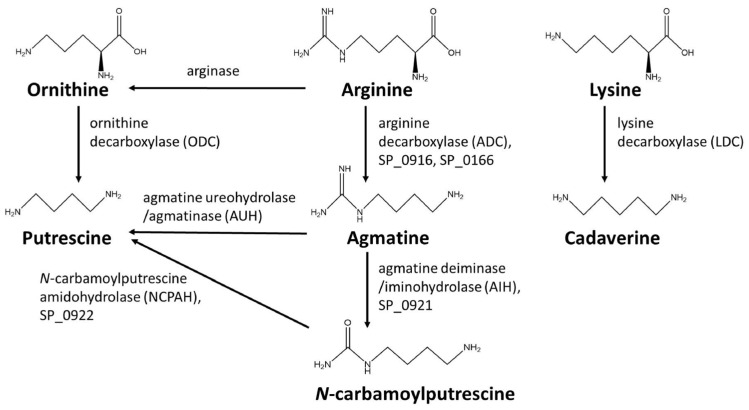
Polyamine biosynthesis pathway substrates and products.

To evaluate the substrate specificity of recombinant SP_0166, arginine, lysine, and ornithine were used as substrates and the reaction products agmatine, cadaverine, and putrescine, respectively, were quantified by LC–MS/MS (Figure 1) [16].

The reaction mixture consisted of 1.163 µmol/L protein, 10 mM substrate, 0.6 mM pyridoxal-phosphate (PLP), 2.5 mM MgSO_4_, and 50 mM Tris-HCl (pH 7.5) in a total volume of 500 µL, and was incubated for 30 min at 37 °C in the dark and stopped by addition of 12.5 µL of 70% (*w*/*v*) perchloric acid. After incubation on ice for 15 min, the mixture was neutralized by the addition of 25 µL of 10 N KOH. The sample was mixed with 1 mL of 1-butanol containing spermidine-d8 as an internal standard, and the butanol layers were separated by centrifugation for 5 min at 16,100× *g*. The extracted organic layer was dried under nitrogen and reconstituted with 100 µL of an acetonitrile: methanol: water (40:40:20) solution. To optimize the enzymatic activity of SP_0166, activity measurements were conducted across a pH range of 5.5 to 8.5 using 50 mM Tris-HCl under the same reaction conditions. The enzyme was incubated with each substrate at the indicated pH for 30 min at 37 °C and the reaction products were detected by LC–MS/MS, as described above. Time course activity assays were performed with arginine and lysine substrates for 120 min at 37 °C and the reaction was terminated at different time intervals. The data are shown as the percentage of maximum activity of enzyme with each substrate relative to the highest amount of polyamine product determined by LC–MS/MS.

### 2.3. LC–MS/MS Analysis of SP_0166 Enzymatic Activity

The polyamines were analyzed on a TSQ Quantum Access triple-quadrupole tandem mass spectrometer (Thermo Fisher Scientific, San Jose, CA, USA) equipped with the Acquity UPLC system (Waters, Milford, MA, USA). Chromatographic separation was carried out using an Acquity UPLC BEH Amide column (2.1 mm × 150 mm, 1.7 μm) coupled with an Acquity UPLC BEH Amide VanGuard Precolumn (2.1 mm × 5 mm, 1.7 μm) at 40 °C using a column oven, and 10 µL of samples were injected. The mobile phases consisted of water with 0.1% *v*/*v* formic acid (A) and acetonitrile with 0.1% *v*/*v* formic acid (B). The gradient condition was 0 min (5% A, 95% B), 1 min (5% A, 95% B), 4 min (30% A, 70% B), 6 min (95% A, 5% B), 6.5 min (95% A, 5% B), 7 min (5% A, 95% B), and 10 min (5% A, 95% B). The total run time was 10 min and the flow rate was 0.3 mL/min. The column eluate was directed into the mass spectrometer using an electrospray ionization interface in positive mode. The MS conditions were set as follows: spray voltage = 3500 V, vaporizer temperature = 350 °C, sheath gas pressure = 25 psi, auxiliary gas pressure = 10 psi, and capillary temperature = 350 °C. Samples were run in selected reaction monitoring (SRM) mode and with precursor-to-product ion transitions of m/z 175.1 → m/z 70.4 for arginine, m/z 147.1 → m/z 84.4 for lysine, m/z 133.1 → m/z 116.1 for ornithine, m/z 131.1 → m/z 72.4 for agmatine, m/z 103.2 → m/z 86.4 for cadaverine, m/z 89.2 → m/z 72.4 for putrescine, and m/z 132.1 → m/z 115.2 for *N*-carbamoylputrescine. Internal standard included spemidine-d8 (m/z 154.2 → m/z 80.4), which was used to normalize the quantified amounts of substrates and products. Scan time was 0.2 s per SRM and the scan width was m/z 0.01. Optimum collision energy and S-lenses conditions were determined for each compound by using Auto-Tune software (Thermo Xcalibur 2.2 SP1.48) for each analyte by post-column infusion of the individual compounds into a 50% A/50% B blend of the mobile phase being pumped at a flow rate of 0.3 mL/min. Xcalibur software (version 2.2 SP1.48) was utilized for data acquisition and processing.

### 2.4. Enzyme Kinetics Analysis

Kinetic parameters of SP_0166 were analyzed by detecting the amount of the product generated over a range of substrate concentrations from 0.01 mM to 30 mM at pH 7.5 for 1 h. For quantification, calibration standards were prepared for agmatine, cadaverine, and putrescine. The nonlinear regression method of the Michaelis–Menten equation with Sigma Plot v.12 was used to estimate the kinetics parameters (*kcat*, *Km*, and *kcat*/*Km*). All experiments were performed with three independent replicates and the values represent the mean ± the standard deviation of three separate measurements.

### 2.5. Enzyme Inhibition Assays

To investigate the effect of decarboxylase inhibitors on SP_0166 activity, DFMO was added to the reaction mixture in a range between 0.1 to100 mM and DFMA between 0.003 to 3 mM. The inhibitors were stored as 2 M stock solutions in water at −20 °C. The stocks were diluted with reaction buffer on the day of the experiment. The inhibitors were prepared by diluting each concentration in water and adding it to the reaction mixture after 5 min preincubation at 37 °C in the dark. A parallel control reaction was incubated without inhibitors. The enzyme and inhibitor were incubated at 37 °C for 1 h and polyamine reaction products were extracted as described earlier. All experiments were performed in triplicate. The concentration of inhibitor that inhibited 50% of the control activity (IC_50_ value) was determined by varying the concentration of the inhibitor DFMA or DFMO and measuring the decarboxylase activity. Sigma Plot v.12 was used to fit the curve through the points and IC_50_ values were interpolated from the fitted curve.

## 3. Results

### 3.1. Expression and Purification of SP_0166

**Figure 2:**
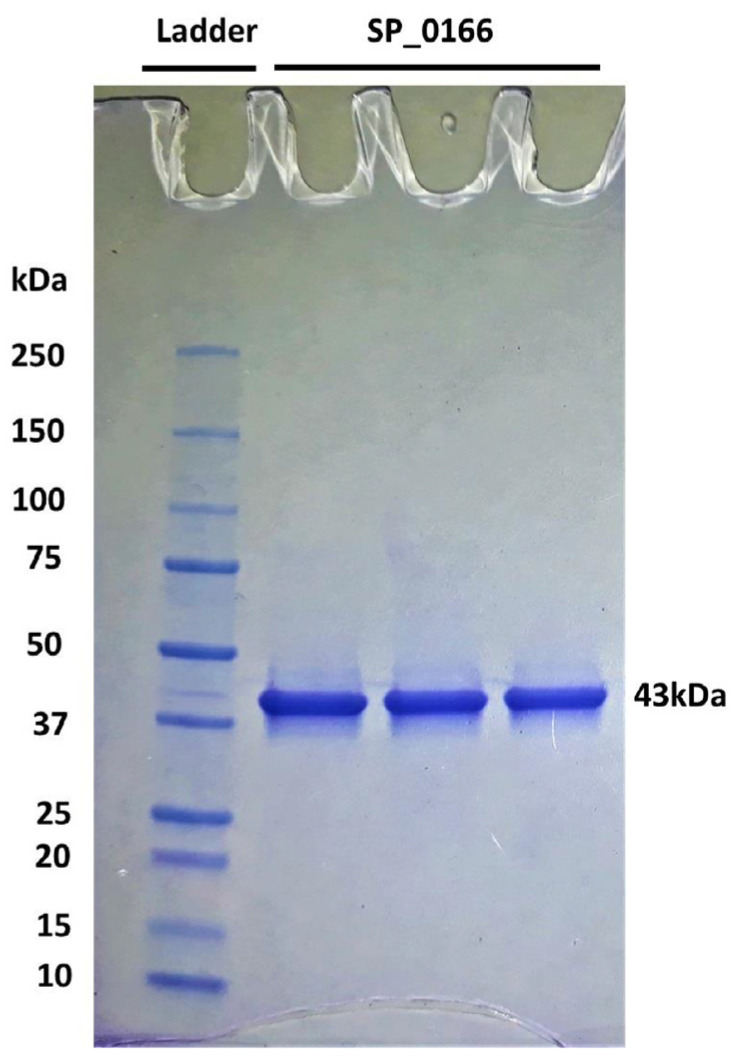
Expression and purification of recombinant SP_0166. The overexpressed and purified recombinant 43 kDa SP_0166 from *S. pneumoniae* TIGR4 was resolved by sodium dodecyl sulfate polyacrylamide gel electrophoresis.

Annotation of *SP_0166* gene in *S. pneumoniae* serotype 4 in bioinformatic databases such as in UniProt, KEGG, and BioCyc [27,28,29] indicates that it is potentially a pyridoxal-dependent decarboxylase with a broad substrate specificity, which includes ornithine, lysine, and arginine. The 368 amino acid protein has a predicted molecular weight of 42,757 Da. Purified recombinant SP_0166 is ~43 kDa, as expected (Figure 2 and Figure S1). The yield of recombinant SP_0166 (4.09 mg protein/100 mL culture) is comparable with previously characterized recombinant SP_0916/arginine decarboxylase (3.42 mg protein/100 mL culture) [13].

### 3.2. Substrate Specificity of SP_0166

**Figure 3:**
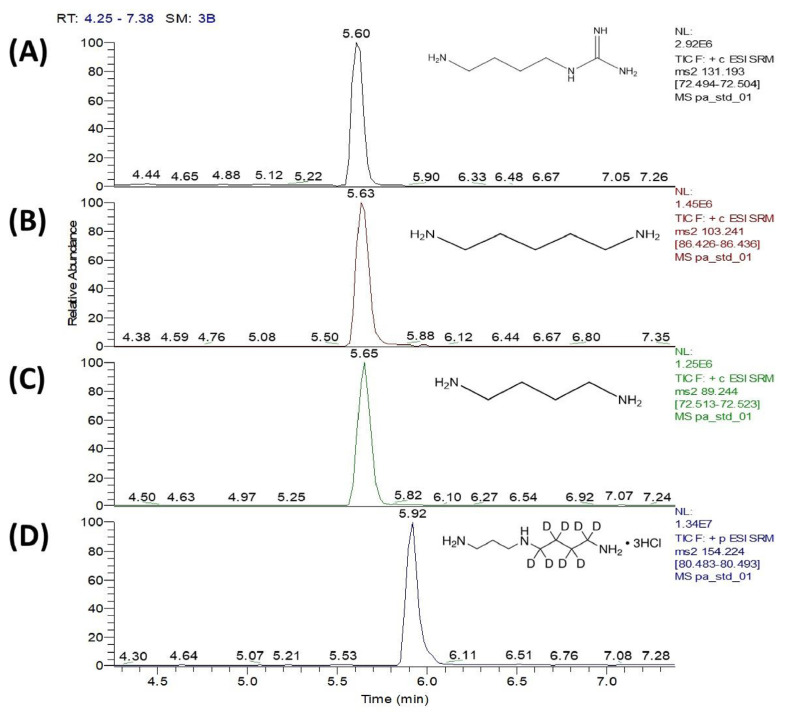
UHPLC–MSMS chromatograms of (**A**) agmatine, (**B**) cadaverine, and (**C**) putrescine, which are the decarboxylation products of arginine, lysine, and ornithine, respectively, and (**D**) spermidine-d8 (internal standard). Four separate single monitoring (SRM) transitions are shown for synthesized polyamine standards within 10 min running time.

To determine the substrate specificity of SP_0166, the recombinant enzyme was incubated with different substrates and the reaction end products were measured. With arginine, lysine, and ornithine as substrates, the corresponding products would be agmatine, cadaverine, and putrescine, respectively, and these were measured by LC–MS/MS. HILIC columns in LC–MS/MS streamline the sample preparation process by eliminating the need for derivatization of the target polyamines. The analysis achieved simultaneous quantification of polyamines within a 10 min run time, and the chromatogram peaks corresponding to the polyamines were detected (Figure 3).

**Figure 4:**
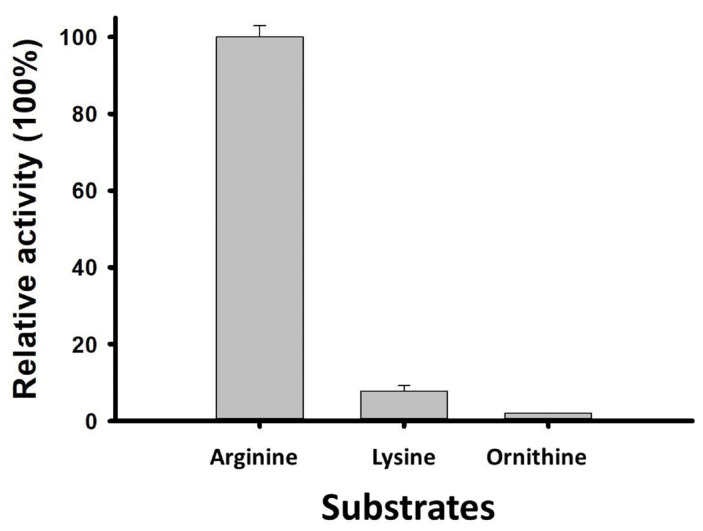
SP_0166 is an arginine decarboxylase. SP_0166 functions as an arginine decarboxylase, demonstrating the highest relative activity with arginine as a substrate. It exhibits relatively lower activity with lysine and negligible activity with ornithine.

Similar to SP_0916, LC–MS/MS analysis of SP_0166 reaction products showed that arginine is the preferred substrate, as the relative activity of the enzyme to produce agmatine, the end product of arginine decarboxylation, was 12-fold higher than for cadaverine, the end product of lysine decarboxylation (Figure 4) [16]. The SP_0166 enzyme in TIGR4 is annotated broadly as a pyridoxal-dependent decarboxylase within the Orn/Lys/Arg family. Our results clearly establish that SP_0166 preferentially decarboxylates arginine and has lower affinity for lysine and ornithine.

### 3.3. Optimization of pH and Incubation Time to Enhance the ADC Activity

**Figure 5:**
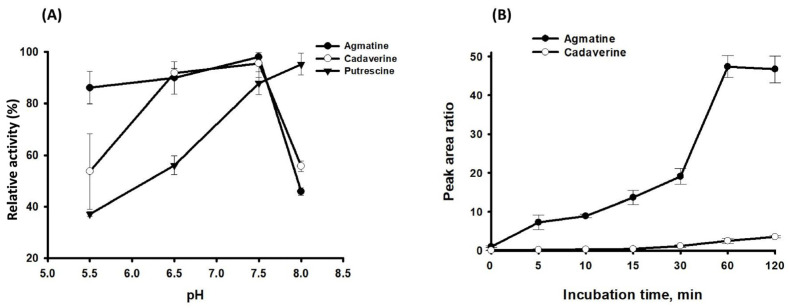
Optimization of reaction conditions for polyamine analysis by LC–MS/MS. Panel (**A**) depicts the impact of pH on the activity of recombinant SP_0166, while panel (**B**) presents the time course of agmatine and cadaverine production from arginine and lysine substrates, respectively.

ADCs in *E. coli*, a model organism for bacterial polyamine metabolic pathways, are annotated to be either constitutive (optimal catalytic activity at pH 5.2) or inducible (optimal pH is 8.23) [30,31,32]. To characterize the enzyme activity of SP_0166, optimal pH was determined. The maximum activity of SP_0166 was found at pH 7.5 and the effect of pH on each substrate is shown in Figure 5A. The enzyme activity increases steadily from pH 6.5 to pH 7.5 and rapidly decreases as pH increases, except when ornithine is the substrate. Generally, the effect of pH on SP_0166 activity with arginine and lysine is similar between pH 6.5–8.0.

We measured the arginine and lysine decarboxylase activity of SP_0166 at pH 7.5 at different time points up to 120 min. As shown in Figure 5B, both agmatine and cadaverine levels gradually increased in 60 min. Agmatine synthesis from arginine increased between 30 min and 60 min and decreased slightly after 60 min.

### 3.4. Enzyme Kinetics Analysis

**Figure 6:**
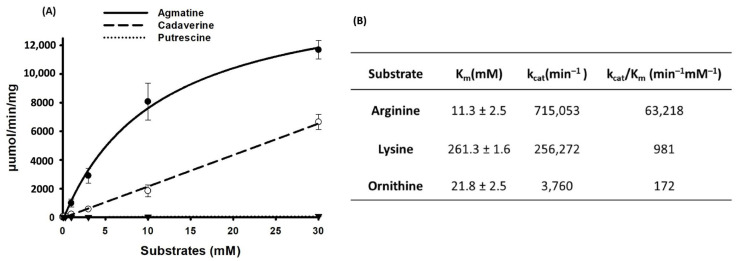
LC–MS/MS analysis of SP_0166 enzyme kinetics. (**A**) Comparison of the enzyme kinetics for the conversion of arginine to agmatine (●), lysine to cadaverine (○), and ornithine to putrescine (▼) at different substrate concentrations. The lines represent curve-fitting for each point. (**B**) Comparison of kinetic parameters. The results shown are the means of triplicate experiments, and data represent mean ± standard deviation.

The enzyme kinetics of the SP_0166 were measured at various concentrations of arginine, lysine, or ornithine that ranged from 0.03 mM to 30 mM. The SP_0166 catalyzed decarboxylation of substrates, and the concentration of each substrate was expressed in the form of the concentration of the converted product, as shown in Figure 6A. The kinetic parameters determined using the Michaelis–Menten equation are summarized in Figure 6B, and goodness of fit (R2) was 0.9962, 0.9998, and 0.9201 for arginine, lysine, and ornithine, respectively. The *Km* for catalysis of the substrate–enzyme pairs was in this order: lysine > ornithine > arginine, demonstrating that SP_0166 is most efficient at decarboxylating arginine. Also, the catalytic efficiency (*kcat*/*Km*) of SP_0166 for the conversion of arginine to agmatine is about 64-fold and 368-fold higher than for the conversion of lysine to cadaverine and ornithine to putrescine, respectively (Figure 6B). These findings unequivocally establish SP_0166 as primarily functioning as an arginine decarboxylase. While SP_0166 exhibits the capacity to decarboxylate lysine and ornithine substrates, it does so with a markedly lower catalytic efficiency.

### 3.5. Inhibition of Decarboxylase Activity by DFMA and DFMO

**Figure 7:**
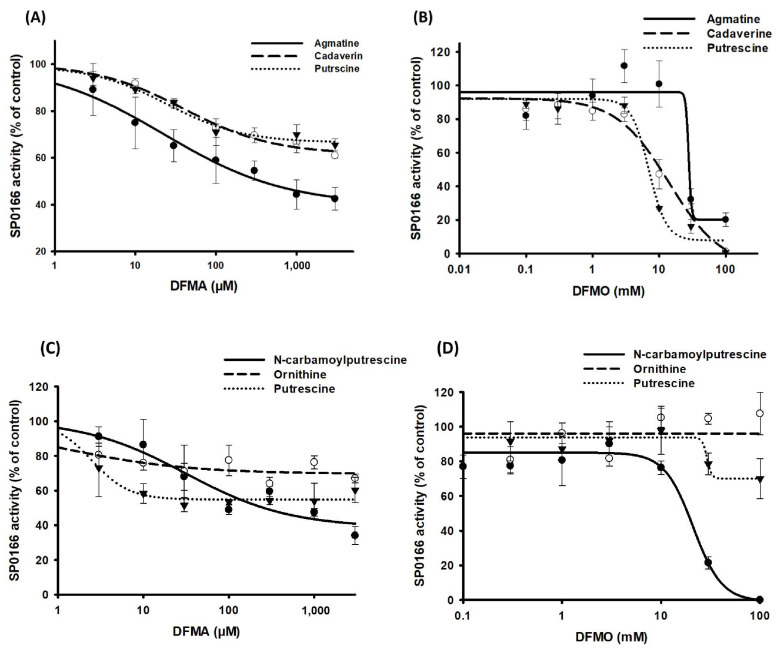
Inhibition of recombinant SP_0166 decarboxylase activity by DFMA and DFMO. Inhibition potency of different concentrations of (**A**) DFMA and (**B**) DFMO on polyamine synthesis from arginine (●), lysine (○), and ornithine (▼) substrates. Inhibition potency of different concentrations of (**C**) DFMA and (**D**) DFMO on synthesis of *N*-carbamoyl putrescine (●), ornithine (○), and putrescine (▼) from arginine. The results shown are the means of triplicate experiments, and data represent mean ± standard deviation.

To study the effects of DFMO and DFMA, the recombinant SP_0166 protein was preincubated with arginine, lysine, and ornithine for 5 min and followed by the addition of each inhibitor at different concentrations and incubation for an additional 1 h. The samples were extracted and analyzed by LC–MS/MS for the polyamine quantitation. Enzyme activity without inhibitor was used for control and relative activity values were calculated for each concentration. Corrected activity was plotted against nominal inhibitor concentration and fitted to determine the concentration of the inhibitor required for half-maximal inhibition of substrate generation (IC_50_) [33,34]. Figure 7A,B and Table 2 show DFMA and DFMO with IC_50_ values in the µM and mM range, respectively, for the inhibition pattern of the polyamines produced when arginine, lysine, and ornithine were used as substrates. The reported information on DFMA and DFMO suggests that these compounds primarily bind to and inhibit ADC and ODC, respectively, and our experimental results align with this observation. When SP_0166 was incubated with arginine and DFMA, the amount of agmatine produced was significantly reduced compared with cadaverine and putrescine. This result indicates that SP_0166 ADC activity is more sensitive to DFMA inhibition than LDC or ODC. In the case of DFMO, agmatine, cadaverine, and putrescine all showed similar inhibition results, showing that SP_0166 enzymatic activity is inhibited by DFMO.

Two distinct enzymes are known to catalyze the conversion of agmatine to putrescine or *N*-carbamoyl putrescine in prokaryotes (Figure 1). Agmatinase and agmatine deiminase convert agmatine to putrescine and *N*-carbamoylputrescine, respectively [6,12,35]. When SP_0166 was incubated with arginine along with DFMA and DFMO, we confirmed that there was a decrease in *N*-carbamoylputrescine levels in a pattern like that of agmatine (Figure 7C,D). Therefore, it could be confirmed that agmatine deiminase was inhibited by DFMA and DFMO. Under the same conditions, the amount of putrescine produced decreased as the concentration of DFMA increased, but there was negligible impact by DFMO. Putrescine is produced from agmatine directly by agmatinase or indirectly via *N*-carbamoylputrescine by *N*-carbamoylputrescine amidohydrolase. Based on our results, *N*-carbamoylputrescine amidohydrolase and agmatine deiminase are affected by DFMA but not by DFMO. Arginase is an enzyme that hydrolyzes arginine to ornithine and urea [36,37]. However, there was no inhibition of ornithine synthesis from arginine, indicating that DFMA had no effect on arginase activity. This result also supports that arginase has not yet been annotated in the TIGR4 genome. Based on these results, it can be expected that agmatine deiminase is affected by both DFMA and DFMO, like ADC, but agmatinase and *N*-carbamoylputrescine amidohydrolase are inhibited only by DFMA.

## 4. Discussion

In numerous human bacterial pathogens, polyamines play a pivotal role in shaping host–pathogen interactions. For instance, pretreatment of eukaryotic cells with cadaverine renders them less susceptible to the impact of *Shigella* enterotoxins [37]. Additionally, cadaverine functions to impede the release of *Shigella flexneri* from the phagocytic vacuole [38]. Notably, polyamines exert significant influence on biofilm synthesis and maturation in various pathogens, including *Yersinia pestis* [39], *Vibrio cholerae* [40], and *Pseudomonas aeruginosa* [41]. Furthermore, polyamines are integral to the survival and colonization strategies employed by *Helicobacter pylori* within the gastric mucosa. Their role extends to facilitating the adaptation of the bacterium to the acidic conditions prevailing in the stomach [42]. Additionally, these versatile molecules contribute significantly to the adaptive processes undertaken by *Salmonella* in the host environment. Polyamines are actively involved in modulating the expression of genes associated with *Salmonella* invasion, intracellular survival, and systemic infection [43].

We and others have shown that polyamine synthesis and transport mechanisms are important for various bacterial responses under different physiological conditions. Specifically, the deletion of the spermidine synthesis has been linked to a delayed onset of autolysis in *S. pneumoniae* [44], while deletion of agmatine biosynthesis genes has been associated with prolonged lag phases during its growth [11,13]. Moreover, the disruption of the polyamine transport, as observed in the transport operon deletion strain *ΔpotABCD*, results in reduced putrescine and spermidine levels, and attenuation in vivo [13,45]. Similarly, deletion of the spermidine biosynthesis impedes the growth of *Campylobacter jejuni* [46], and simultaneous deletion of genes responsible for agmatine and putrescine production leads to growth impairment in *Pseudomonas aeruginosa* [35]. Furthermore, mutations in spermidine biosynthesis genes render *P. aeruginosa* more susceptible to antibiotics and oxidative stress [47]. Most importantly, deletion of either the polyamine transport operon or the gene involved in agmatine production in *S. pneumoniae* results in the loss of capsule [11,12,13], the predominant virulence factor in pneumococci, which explains the reported in vivo attenuation.

To understand the contribution of individual and combined effects of genes of the polyamine biosynthesis pathway on pneumococcal metabolism and virulence, it is critical to accurately annotate the function of genes from this pathway. The current annotation of the polyamine biosynthesis pathways in the pneumococci is limited and could be inaccurate (as shown with the annotation of *cadA* in our previous work [11,13]). In the genome annotation of *S. pneumoniae* TIGR4, the open reading frame SP_0166 is annotated to be a pyridoxal-dependent decarboxylase within the Orn-Lys-Arg family. This study establishes that SP_0166 is an arginine decarboxylase (ADC) that catalyzes agmatine synthesis and shows the inhibition of polyamine synthesis by DFMA and DFMO using a relatively sensitive LC–MS/MS approach.

Compared to the PLP-dependent inducible and constitutive arginine decarboxylases (ADC) in *E. coli*, which require optimum pH values of 5.2 and 8.4, respectively, for catalytic activity and *Paramecium bursaria* chlorella virus-1 with an optimum pH of 8.2 [48], the optimum pH for SP_0166 is 7.5. The *Km* values of ADCs from *E. coli* and chlorella virus-1 range between 0.03 to 0.65 mM. Despite a higher *Km* value for arginine (11.3 mM), catalytic efficiency (~1.05 × 10^6^ s^−1^ M^−1^) of SP_0166 (adapted from Figure 6) is comparable to that of *E. coli* ADC (1.1 × 10^6^ s^−1^ M^−1^), and is higher than that of *Paramecium bursaria* chlorella virus-1 (3.3 × 10^4^ s^−1^ M^−1^). Interestingly, while optimum pH of *Shewanella algae* ADC is 7.5 and its *Km* is 14.55 mM [49], like SP_0166, its catalytic efficiency of 8.67 × 10^2^ s^−1^ M^−1^ is significantly lower compared to SP_0166 (1.05 × 10^6^ s^−1^ M^−1^).

In the previous study, we demonstrated SP_0916 (~54 kDa) in pneumococcal serotype 4 to be an ADC [13]. The discovery here, that SP_0166 is also an ADC, is intriguing but not entirely surprising, considering that recent reports on convergent evolution have revealed the emergence of ADCs from at least four different protein folds. Two forms of ADC are pyridoxal 5′-phosphate (PLP)-dependent enzymes, while the other two utilize pyruvoyl cofactors [50]. However, this means that the gene *cadA*, which encodes an enzyme for the biosynthesis of cadaverine, is still lacking in the pneumococcal genomes. Moreover, the current annotation of polyamine metabolism in the pneumococcal genome shows other notable deficiencies. ODC, a major biosynthetic enzyme that catalyzes the conversion of ornithine to putrescine, is not annotated in pneumococcal genomes in bioinformatic databases. Interestingly, ODC in Ca. *Pelagibacter ubique* and Ca. *Fonsibacter ubiquis* have been shown recently to evolve into ADC [51,52], which could additionally explain our discovery of two different ADCs in pneumococcus. Information on polyamine acetyltransferases that regulate motility and biofilm formation [53], and deacetylases that utilize acetylated polyamine substrates, is also poorly described [6]. Enzymes of the pyridoxal-dependent decarboxylase family, which catalyze the synthesis of polyamines, are expected to potentially utilize a variety of substrates amino acids. Characterizing SP_0166 as an ADC within the pyridoxal-dependent decarboxylase family here has significantly enhanced the annotation of the polyamine synthesis pathway in Spn TIGR4.

Agmatine produced by ADC is an intermediate in the synthesis of putrescine and spermidine and plays an important role in regulating CPS in pneumococci [13]. We already reported that DFMO inhibits CPS in multiple pneumococcal serotypes, and our results demonstrate that DFMO could inhibit ADC activity and play a prominent role in elucidating mechanisms related to altered polyamine synthesis and CPS inhibition [16]. Building upon our previous findings with SP_0916/ADC and SP_0166 in this work, future studies will focus on characterizing the role of SP_0166 in pneumococcal growth in vitro, its impact on the expression of other genes and proteins, its influence on the response to stressors, and potential impact on CPS, and, ultimately, its contribution to virulence in vivo. These studies will facilitate direct comparisons of the significance and roles of the two established ADCs in *S. pneumoniae*, which are beyond the scope of this study.

In conclusion, polyamine biosynthesis and transport genes are highly conserved across various pneumococcal serotypes. Therefore, understanding the polyamine–CPS nexus could help identify new serotype-independent vaccine antigens. Thus, a comprehensive understanding of the polyamine metabolic pathway can provide new approaches for the prevention and treatment of pneumococcal infection.

## Figures and Tables

**Table 1:** Primers used for PCR amplification of SP_0166 from *S. pneumoniae*.

Primer	Sequence
SP_0166-F BamHI	AATTGGATCCATTAATAAAAAAATACAACAAGTTGTTTTGGAATCATTACAG
SP_0166-R XhoI	AATTCTCGAGATATGTCAAGTTTTTTGTCCACAAATATACCTCCC

Sequences complementary to restriction sites are underlined.

**Table 2:** Inhibition potency of DFMO and DFMA against enzymatic activity of SP_0166 with different substrates.

Substrate	Polyamine	DFMO IC_50_ (mM)	DFMA IC50 (µM)
Arginine	Agmatine	27.0 +/− 4.5	21.6 +/− 10.4
	*N*-carbamoyl putrescine	23.3 +/− 4.8	35.7 +/− 12.3
Lysine	Cadaverine	15.4 +/− 3.8	42.3 +/− 13.2
Ornithine	Putrescine	11.5 +/− 4.4	30.9 +/− 14.9

Data represent the mean ± standard deviation of triplicate experiments.

## Data Availability

Data are contained within the article.

## Supplementary Materials

The following supporting information can be downloaded at: https://www.mdpi.com/article/10.3390/biom14040463/s1, Figure S1: Expression and purification of recombinant SP_0166 resolved by sodium dodecyl sulfate polyacrylamide gel electrophoresis.
