# Supplementary material for: Characterization of an Arginine Decarboxylase from Streptococcus pneumoniae by Ultrahigh-Performance Liquid Chromatography–Tandem Mass Spectrometry
Source: Biomolecules. 2024 Apr 10;14(4):463. doi: 10.3390/biom14040463 (PMC11048482; doi:10.3390/biom14040463)
Supplement: Supplementary file 1 [file biomolecules-14-00463-s001.zip › biomolecules-2944760-supplementary.pdf]

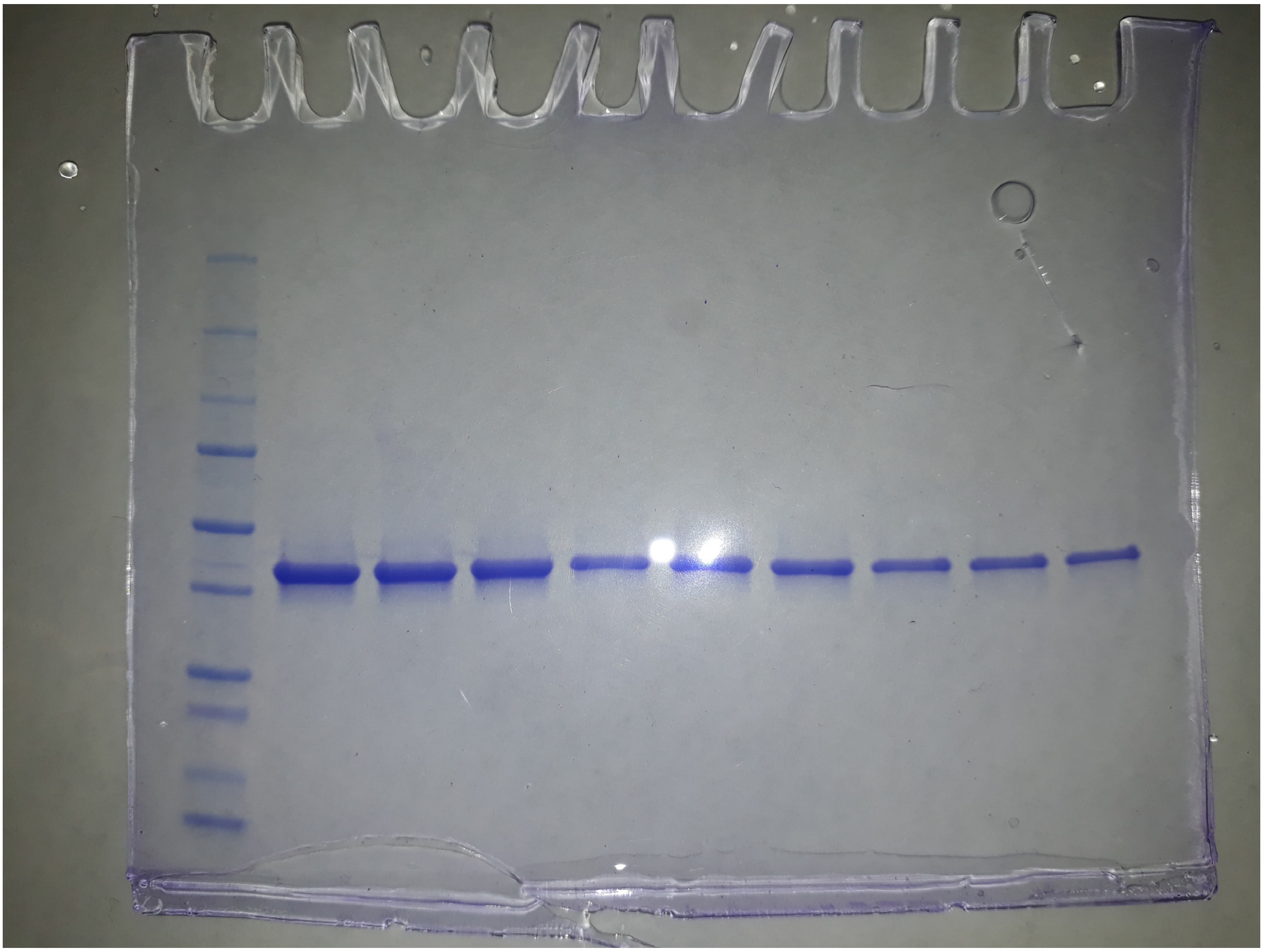

Figure S1. Expression and purification of recombinant SP_0166 resolved by sodium dodecyl sulfate polyacrylamide gel electrophoresis.
